# Use of Tamoxifen Derivatives in Combination with Colistin against Colistin-Resistant *Acinetobacter baumannii*

**DOI:** 10.1021/acsomega.6c05050

**Published:** 2026-10-07

**Authors:** Irene Molina Panadero, Angela Rey Hidalgo, Isabella Rimoldi, Giorgio Facchetti, Michael S. Christodoulou, Younes Smani

**Affiliations:** † Centro Andaluz de Biología del Desarrollo, Universidad Pablo de Olavide/Consejo Superior de Investigaciones Científicas/Junta de Andalucía, Sevilla 41013, Spain; ‡ Department of Pharmaceutical Sciences (DiSFarM), Università degli Studi di Milano, Via Golgi 19, 20133 Milano, Italy; § Department of Food, Environmental and Nutritional Sciences (DeFENS), University of Milan, Via Celoria 2, 20133 Milan, Italy; ∥ Departamento de Biología Molecular e Ingeniería Bioquímica, 16772 Universidad Pablo de Olavide, Sevilla 41013, Spain

## Abstract

Drug repurposing has led to the identification of tamoxifen, a well-known chemotherapy drug, as a compound with relevant antibacterial properties. In this field, the development of new tamoxifen derivatives with antimicrobial activity, particularly against colistin-resistant *Acinetobacter baumannii*, represents an important area of research. We evaluated the MICs of 22 and 3 tamoxifen derivatives against wild-type and colistin-resistant *A. baumannii* strains, respectively, using microdilution assays, either as monotherapy or in combination with colistin. While all derivatives showed MIC values above 64 mg/L when tested as monotherapy, combining derivatives **1**, **14**, and **18** with colistin reduced tamoxifen derivatives’ MICs to 0.125–2 mg/L for the reference strain ATCC 17978 and to 0.5–1 mg/L for the colistin-resistant Ab22 strain. Growth assays showed that these derivatives delayed Ab22 proliferation during the first 4 h. Moreover, these derivatives increased membrane permeabilization without altering the expression of the outer membrane proteins (OMPs). These findings suggest that tamoxifen derivatives in combination with colistin exhibit antibacterial activity against a colistin-resistant *A. baumannii*, potentially broadening the spectrum of available drug treatments for combating MDR *A. baumannii*.

## Graphical abstract

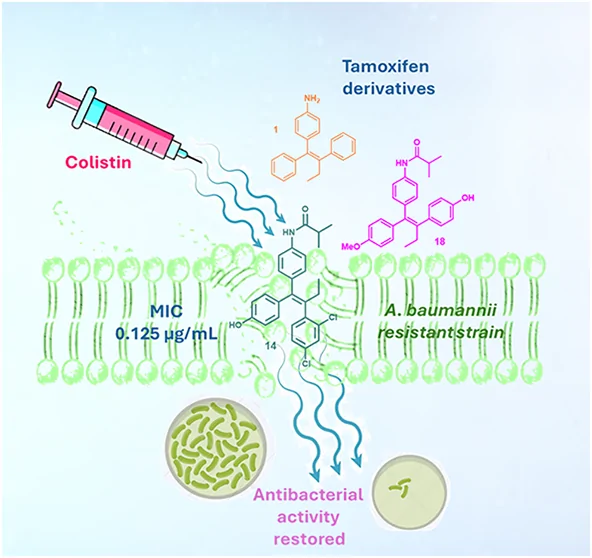

## Introduction

Over the past few decades, the emergence and spread of antimicrobial resistance among Gram-negative bacilli (GNB) have become a major global health concern. This phenomenon is supported by multiple adaptive mechanisms operating at the genomic, transcriptomic, and proteomic levels.^1–5^ At the same time, the development of novel antibiotics has markedly slowed, raising concerns about the advent of a so-called “Post-Antibiotic Era”.^6^ These challenges have prompted the search for alternative therapeutic options, including nonantibiotic antimicrobial agents and the repurposing of approved drugs. Such compounds may be used either alone or in combination with the limited number of clinically effective antibiotics currently available.^7–9^

Drug repurposing offers a particularly appealing strategy because it may shorten development timelines by exploiting the known pharmacological and safety profiles of existing medicines. Tamoxifen, a selective estrogen receptor modulator extensively used in the treatment of estrogen receptor-positive breast cancer and its metastatic forms, represents a notable example. In addition to its established anticancer activity, tamoxifen has recently shown antibacterial effects against several clinically relevant pathogens, including the Gram-positive species *Staphylococcus epidermidis* and *Enterococcus faecalis* and the Gram-negative species *Escherichia coli* and *Acinetobacter baumannii*. Tamoxifen is metabolized by cytochrome P450 enzymes into three major derivatives: *N*-desmethyltamoxifen, 4-hydroxytamoxifen, and endoxifen (Figure 1).^9–13^ Although their antimicrobial potential remains relatively underexplored, a small number of studies have investigated the activity of these metabolites against different infectious agents.^10,15–20^

**Figure 1:**
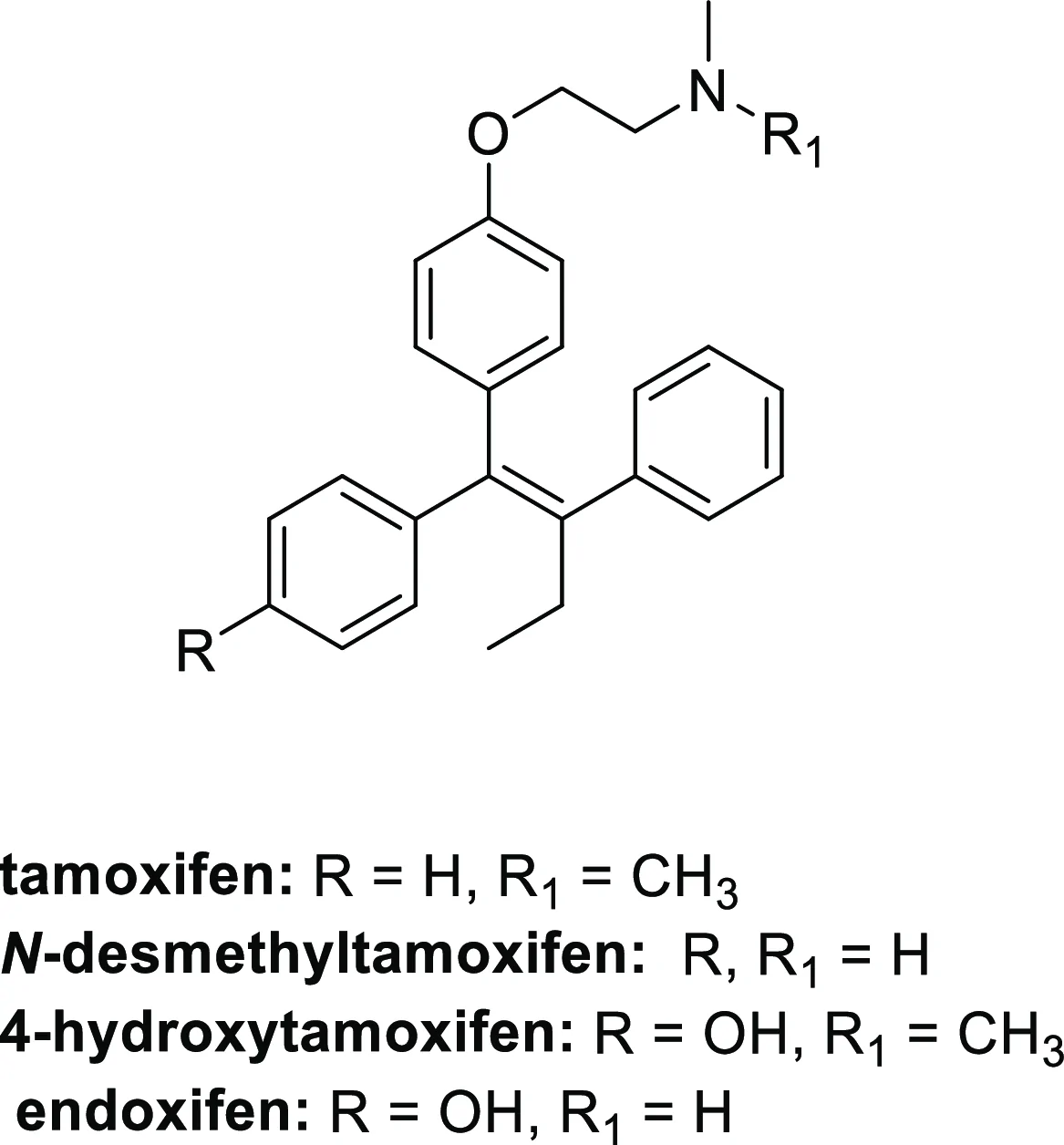
Structures of tamoxifen, *N*-desmethyltamoxifen, 4-hydroxytamoxifen and endoxifen.

In particular, 4-hydroxytamoxifen is a weak base that has been reported to protect cultured cells and mice from the lethal effects of Shiga toxins 1 and 2 (STx1 and STx2).^16^ Activity against *Plasmodium falciparum*, *Cryptococcus neoformans*, and *Mycobacterium tuberculosis* has also been described, with MIC_50_ values of approximately 2.5–5 mg/L against *M. tuberculosis*.^18,20^ Endoxifen, the major active metabolite of tamoxifen, exhibited an MIC of 4 mg/L against *C. neoformans*.^19^ Furthermore, the combination of *N*-desmethyltamoxifen and 4-hydroxytamoxifen showed MIC_50_ values of 8 and 16 mg/L against clinical isolates of *A. baumannii* and *E. coli*, respectively, and of 1 and 2 mg/L against *S. epidermidis* and *E. faecalis*, respectively.^10,11^

Among Gram-negative pathogens, multidrug-resistant *A. baumannii* is particularly difficult to treat. The progressive loss of effective therapeutic options has increased reliance on colistin, which has traditionally been reserved for severe infections caused by multidrug-resistant bacteria. However, its growing clinical use has been accompanied by the widespread emergence of colistin-resistant strains over the past decade.^21,22^

## Results and Discussion

Based on the reported antibacterial activity of tamoxifen and its metabolites *N*-desmethyltamoxifen, 4-hydroxytamoxifen and endoxifen against *A. baumannii*,^10,23^ we hypothesized that tamoxifen derivatives might enhance antimicrobial activity against resistant strains, alone or in combination with colistin. To identify new chemical compounds with antimicrobial potential, we evaluated a library of 22 tamoxifen derivatives, previously described for their antibacterial activity against methicillin-resistant *S. aureus*,^9^ against both colistin-susceptible and colistin-resistant *A. baumannii* (Table 1).

**Table 1:**
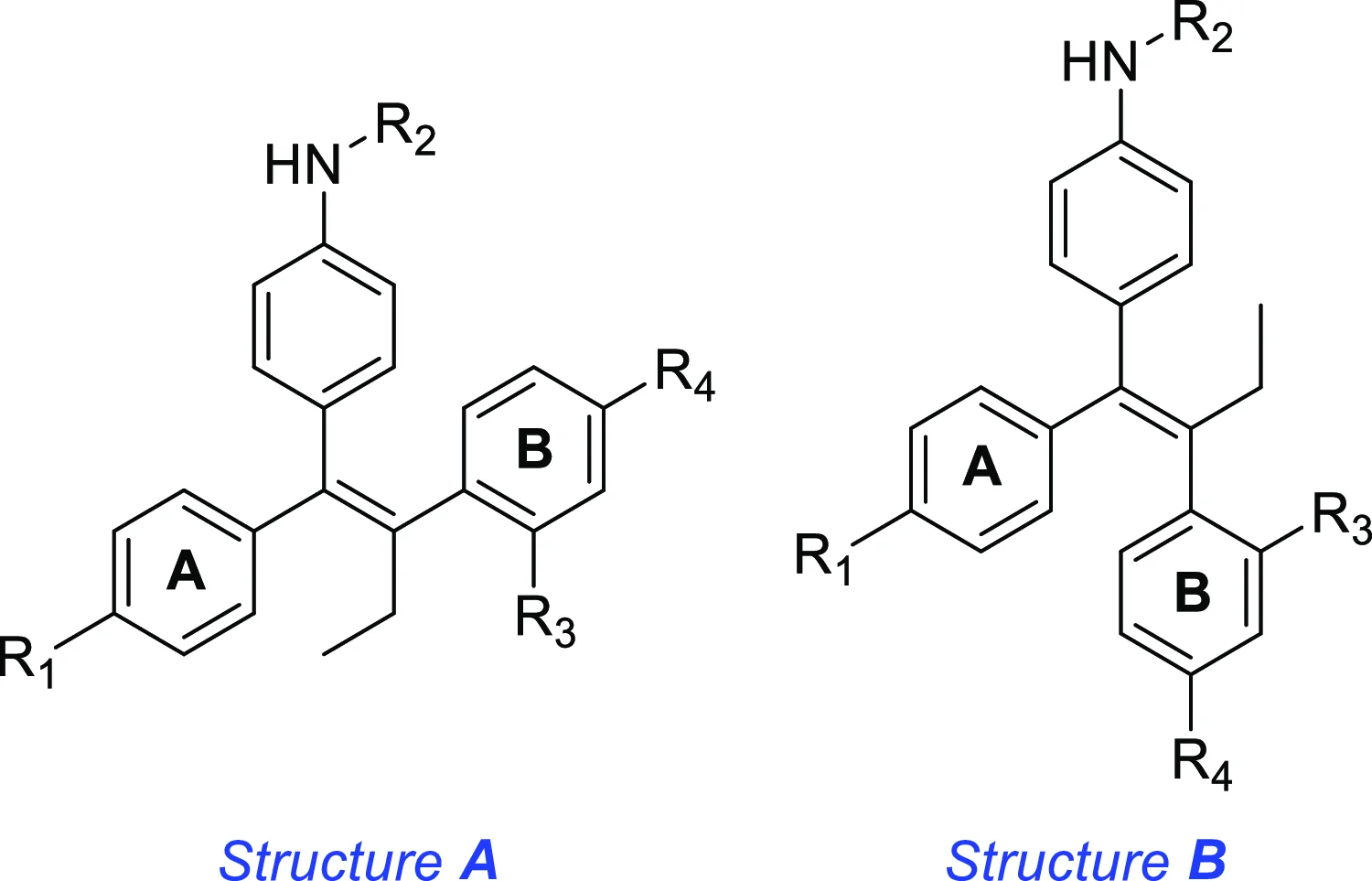
MIC (mg/L) of Tamoxifen Derivatives 1–22 in Monotherapy and in Combination with Colistin for Reference Strain of *A. baumannii*

comp.	structure	R_1_	R_2_	R_3_	R_4_	*A. baumannii* ATCC 17978	*A. baumannii* ATCC 17978 with colistin^a^
**1**	A	H	H	H	H	>64	0.5–1
**2**	A	OH	CON(CH_2_CH_3_)_2_	H	OH	>64	>64
**3**	A	OBn	COCH_2_CH_3_	H	OBn	>64	>64
**4**	B	OBn	COCH_2_CH_3_	H	OBn	>64	>64
**5**	A	OH	CON(CH_3_)_2_	H	OH	>64	>64
**6**	B	OH	CON(CH_3_)_2_	H	OH	>64	>64
**7**	A	OCH_3_	CO*i*Propyl	H	H	>64	>64
**8**	B	OCH_3_	CO*i*Propyl	H	H	>64	>64
**9**	A	H	CO*i*Propyl	H	H	>64	>64
**10**	B	H	CO*i*Propyl	H	H	>64	>64
**11**	B	H	CO*i*Propyl	H	OBn	>64	>64
**12**	A	H	CO*i*Propyl	H	OBn	>64	>64
**13**	A	OH	CO*i*Propyl	Cl	Cl	>64	>64
**14**	B	OH	CO*i*Propyl	Cl	Cl	>64	2
**15**	A	OCH_3_	CO*i*Propyl	H	OBn	>64	>64
**16**	B	OCH_3_	CO*i*Propyl	H	OBn	>64	>64
**17**	B	OCH_3_	CO*i*Propyl	H	OH	>64	>64
**18**	A	OCH_3_	CO*i*Propyl	H	OH	>64	0.125–0.5
**19**	A	OH	CO*i*Propyl	H	H	>64	>64
**20**	B	OH	CO*i*Propyl	H	H	>64	>64
**21**	A	OBn	CO*i*Propyl	H	OBn	>64	>64
**22**	B	OBn	CO*i*Propyl	H	OBn	>64	>64
**Tam**						>256 ^10^	

a Colistin concentration: 0.125 mg/L. TAM: tamoxifen.

The selected compounds did not exhibit antibacterial activity as monotherapy (MICs > 64 mg/L) against the reported strains. However, as previously observed for other repurposed drugs,^24–26^ several compounds exhibited antibacterial activity against colistin-resistant *A. baumannii* when combined with sub-MIC concentrations of colistin (Table 1). In particular, tamoxifen derivatives, **1**, **14** and **18**, showed markedly reduced MIC values (0.125–2 mg/L) when combined with sub-MIC colistin (Table 1).

Structure–activity relationship (SAR) analysis highlighted several key trends among both structure A and structure B derivatives. In detail, analogues bearing electron-donating substituents on one or both phenyl rings A and B were inactive (compounds **3**, **4**, **7**, **8**, **11**, **12**, **15**, **16**, **21** and **22**), as were those containing only a hydroxyl group on one or both phenyl rings A and B (compounds **2**, **5**, **6**, **19** and **20**). However, it should be mentioned that compounds **2**, **5** and **6** bearing hydroxyl groups at position 4 on both phenyl rings, were active against the Gram-positive *S. aureus* (Table 1).^9^ Derivatives bearing a hydroxyl substituent on one of the two phenyl rings A or B and either an electron-withdrawing or an electron-donating group on the other phenyl ring (compounds **14** and **18**) showed activity, suggesting a synergistic contribution of substituents on both rings. These findings suggest that stereochemical considerations and the dynamic formation of active species are key factors in modulating biological or chemical activity.^27–29^ Stereochemistry also plays a role, as evidenced by the comparison between compounds **13** and **14**, where, in the presence of electron-withdrawing substituents (–Cl), the *Z-*isomer (compound **14**, structure B) is active, in contrast to compounds **17** and **18**, in which in the presence of an electron-donating group –OMe, the *E*-isomer (compound **18**, structure A) is active, respectively. In the absence of substituents on both aromatic rings A and B, only the compound bearing a free amino group (compound **1**) was active, whereas derivatives bearing an amide side chain (compounds **9** and **10**) were inactive. The primary amino group appears to be a key determinant of permeation across the Gram-negative bacterial cell envelope. Upon protonation, it can establish favorable hydrogen-bonding interactions that facilitate translocation through the bacterial barrier. These findings provide a clear design rationale for future compound libraries, highlighting the importance of maintaining the primary amino group unmodified in order to preserve its beneficial contribution to bacterial uptake and, ultimately, antibacterial activity.^30–32^

The most promising derivatives, identified in the initial screening by exhibiting MIC ≤ 64 mg/L in combination with colistin (Table 1), were further tested against seven clinical isolates of colistin-resistant *A. baumannii* to assess their antimicrobial potential activity against resistant strains. Tamoxifen derivatives **1**, **14** and **18**, representing different structural modifications, demonstrated enhanced antibacterial activity against the colistin-resistant Ab22 strain when combined with sub-MIC concentrations of colistin, with MIC values ranging from 0.125 to 2 mg/L, whereas the other colistin-resistant isolates did not show the same susceptibility, and tamoxifen alone or in combination with colistin has MIC > 64 mg/L. These findings suggest that the observed synergistic effect is associated with different chemical scaffolds rather than being restricted to a single derivative and is probably influenced by genetic background and resistance profile of the isolates. Further genomic and molecular studies are required to identify the determinants underlying the observed synergism. Of note, the same three derivatives showed MICs > 64 mg/L when tested as monotherapy (Table 2). Furthermore, treatment with tamoxifen derivatives **1**, **14** and **18** at the synergistic concentrations identified (0.125–2 mg/L) did not significantly affect the viability of A549 cells, which remained between 90.1% and 100%, supporting the favorable safety profile of these compounds at therapeutically relevant concentrations.

**Table 2:** MIC (mg/L) of Tamoxifen Derivatives 1, 14, and 18 in Monotherapy and in Combination with Colistin for Clinical Colistin-Resistant *A. baumannii*

		**1**		**14**		**18**	
strain	colistin	w/o colistin	with colistin^a^	w/o colistin	with colistin^a^	w/o colistin	with colistin^a^
**RC-64**	32	>64	>64	>64	>64	>64	>64
**Ab11**	256	>64	>64	>64	>64	>64	>64
**Ab20**	64	>64	>64	>64	>64	>64	>64
**Ab21**	128	>64	>64	>64	>64	>64	>64
**Ab22**	128	>64	0.5–1	>64	0.125	>64	0.5–1
**Ab99**	64	>64	>64	>64	>64	>64	>64
**Ab113**	64	>64	>64	>64	>64	>64	>64

a Colistin concentration: 0.125 mg/L. w/o: without.

The synergistic interaction was confirmed by calculating the Fractional Inhibitory Concentration index (FICI) for the Ab22 strain. For the combination of colistin and derivative **18**, the FICI index was 0.26 (calculated as the ratio of the MIC in combination to the MIC alone), which, according to the standard criteria,^25^ indicates a strong synergistic effect rather than a merely additive interaction. Similar synergistic effects were obtained for derivatives **1** and **14** when combined with colistin being FICI = 0.26 and 0.27, respectively. This finding is particularly relevant because colistin is an important last-resort antibiotic for the treatment of infections caused by MDR Gram-negative bacteria. However, its clinical use is limited by toxicity concerns and the emergence of colistin resistance.^33^ The ability of these derivatives to enhance the efficacy of colistin may allow the use of lower concentrations of colistin while improving antibacterial activity, which could represent a valuable strategy to extend the therapeutic potential use of this antibiotic.

Additionally, these tamoxifen derivatives were also tested against *E. coli* in monotherapy. Further studies using a broader range of colistin-resistant clinical isolates of Gram-negative pathogens, such as *Klebsiella pneumoniae* and *Pseudomonas aeruginosa*, would be necessary to fully evaluate the scope of this synergistic strategy across different bacterial species and to validate the general applicability of this combination strategy.

The antibacterial activity of tamoxifen derivatives **1**, **14**, and **18**, either alone or in combination with colistin, was evaluated against the *A. baumannii* Ab22 strain using bacterial growth curve assays. These assays confirmed that colistin potentiated the antibacterial activity of these derivatives, providing dynamic evidence of their synergistic effect. The combination treatments significantly delayed the growth of the Ab22 strain during the first 4 h compared with monotherapies, although regrowth occurred by 12 h (Figure 2A). This regrowth could be consistent with the emergence of a resistant subpopulation. However, the experiments performed in this study were not designed to determine the underlying mechanism of regrowth, and no susceptibility testing or genomic analyses were conducted to confirm the development of resistance. In addition, other mechanisms such as the regrowth of tolerant or persister cells, adaptive physiological responses, or a decline in the effective antimicrobial concentration over time may also explain this phenomenon.

**Figure 2:**
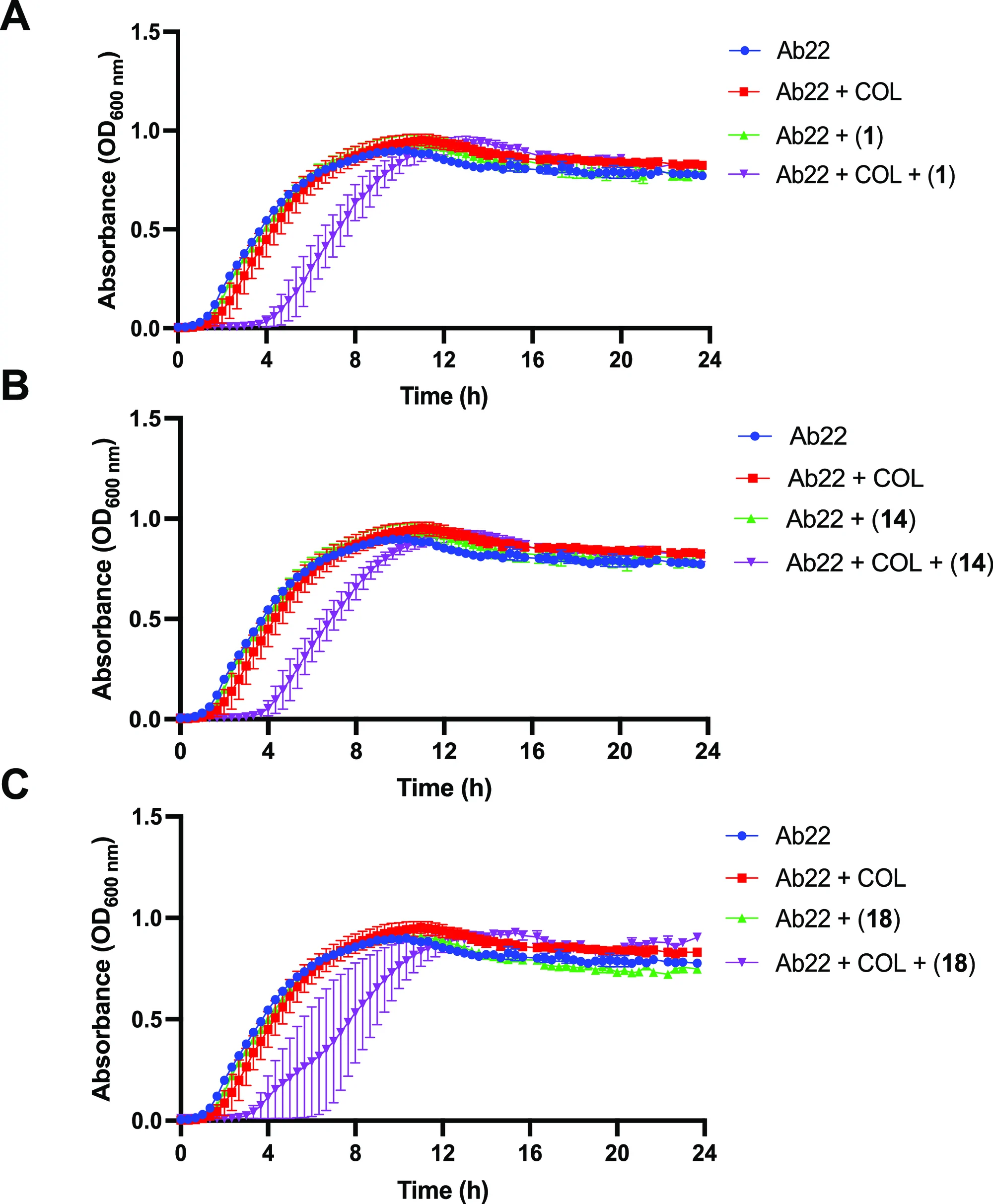
Antibacterial activity of tamoxifen derivatives **1**, **14** and **18** at fixed concentrations and in combination with colistin against clinical colistin-R *A. baumannii*. (A) Bacterial growth curves of *A. baumannii* Ab22 strain in the presence of sub-MIC of colistin (0.25 mg/L), 4 mg/L of tamoxifen derivative **1** and combined treatments for 24 h. (B) Bacterial growth curves of *A. baumannii* Ab22 strain in the presence of sub-MIC of colistin (0.25 mg/L), 4 mg/L of tamoxifen derivative **14** and combined treatments for 24 h. (C) Bacterial growth curves of *A. baumannii* Ab22 strain in the presence of sub-MIC of colistin (0.25 mg/L), 4 mg/L of tamoxifen derivative **18** and combined treatments for 24 h. Data are represented as the mean from two independent experiments. COL: colistin.

While a formal time-kill assay would provide CFU quantification, these growth kinetics demonstrate a strong initial delay exerted by the combination.^25^ In contrast, neither tamoxifen derivative **1** nor colistin affected bacterial growth when administered alone at sub-MIC concentrations. Similar results were observed for derivatives **14** and **18** (Figure 2B,C), confirming that colistin enhanced their antibacterial activity by extending the lag phase and suppressing early exponential growth. Future studies should include time-kill assays, antimicrobial susceptibility testing, and molecular characterization of regrown populations to further characterize the mechanism underlying the regrowth and the bacteriostatic or bactericidal nature of these compounds.

To investigate effect of derivatives **1**, **14** and **18** in combination with colistin on the membrane permeability of the Ab22 strain was examined using ethidium homodimer-1, a fluorescent probe known to enter bacterial cells with compromised membranes.

Fluorescence assays using ethidium homodimer 1 demonstrated significant membrane permeabilization only in the combined treatment samples, supporting this mechanism of action (Figure 3). Since this fluorophore labels only cells with compromised cytoplasmic membranes, these results confirm a loss of cell envelope integrity. Specifically, this synergistic effect likely involves a sequential disruption mechanism: colistin would first destabilize the outer membrane by displacing divalent cations from lipopolysaccharides (LPS),^22^ thereby allowing the hydrophobic tamoxifen derivatives to reach and disrupt the phospholipid-rich inner membrane.^10,25^ This coordinated attack on both membrane layers would explain the potent bactericidal synergy observed.

**Figure 3:**
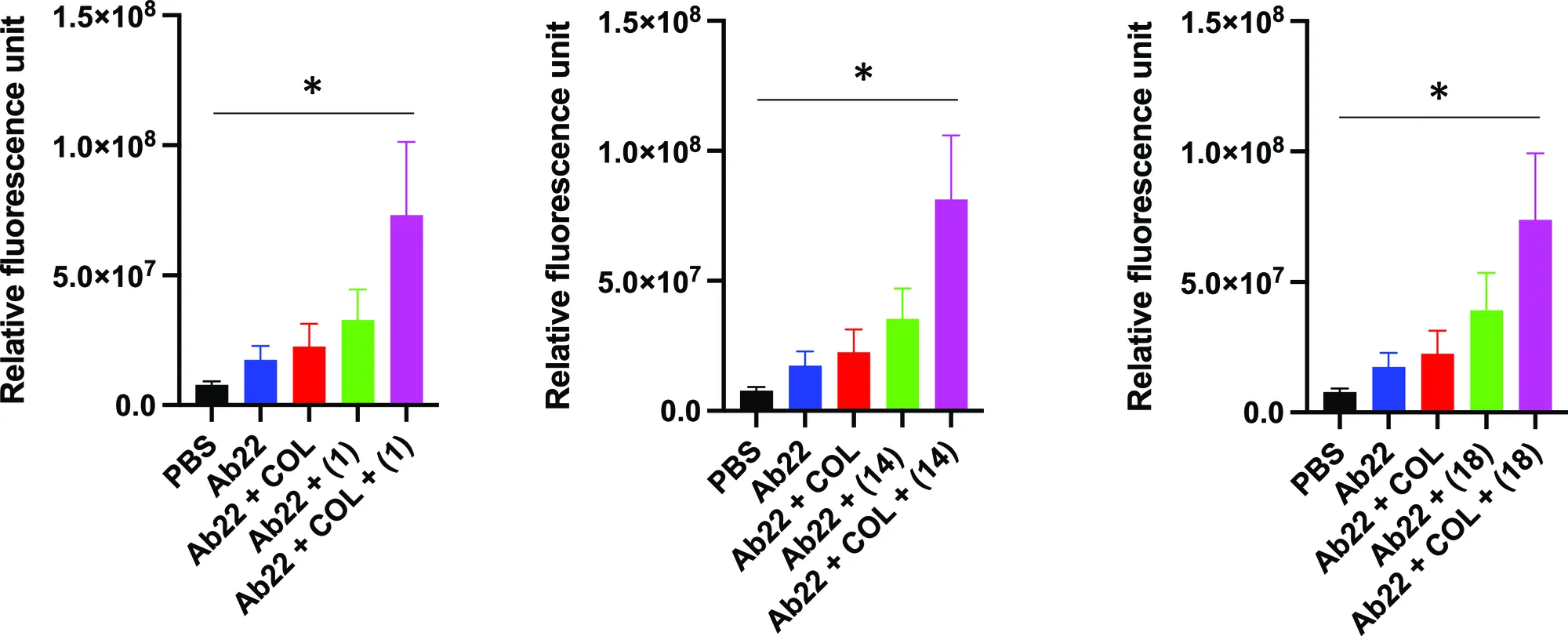
Effect of tamoxifen derivatives **1**, **14** and **18** on the bacterial permeability against clinical colistin-resistant *A. baumannii.* The membrane permeabilization of colistin-resistant *A. baumannii* Ab22 strain in the absence and presence of 4 mg/L of tamoxifen derivatives **1**, **14** and **18** and sub-MIC of colistin (0.125 mg/L) individually, and in combination, incubated for 10 min, was quantified by Typhon Scanner. Data are represented as mean ± SEM from three independent experiments. *P < 0.05 treated cells with combined treatment compared with individual treatment. COL: colistin.

In contrast, treatment with sub-MIC concentrations of tamoxifen derivatives **1**, **14**, or **18**, as well as colistin alone, did not increase membrane permeability in the Ab22 strain. These findings indicate that colistin enhances the membrane-permeabilizing activity of these derivatives against the colistin-resistant *A. baumannii* strain investigated.

The outer membrane protein (OMP) profiles of the Ab22 strain, which serve to assess potential alterations in the bacterial outer membrane, revealed no detectable changes in OMPs binding patterns between control and treatment conditions after 12 h of incubation with sub-MIC levels of colistin and tamoxifen derivatives **1**, **14** and **18** in the expression of the major high molecular weight porins (Figure 4). These results suggest that the derivatives do not alter the primary structural integrity of the OMP expression. Similar observations were reported by Miró-Canturri et al. for tamoxifen metabolites.^10^ However, in the range of 15–20 kDa, subtle variations can be observed. Specifically, derivative **14** (a *Z*-isomer with chlorine substituents) showed a slightly different profile compared with derivative **18** (an *E*-isomer bearing a methoxy group),^32^ suggesting that their distinct chemical structures and stereochemistry may influence the lipid–protein interface or the stabilization of small membrane lipoproteins differently. While these minor differences in the 15–20 kDa range, potentially involving small porins such as the OmpW family, do not indicate a global collapse of the OMP profile, they may contribute to the variations in synergistic activity observed among the derivatives.

**Figure 4:**
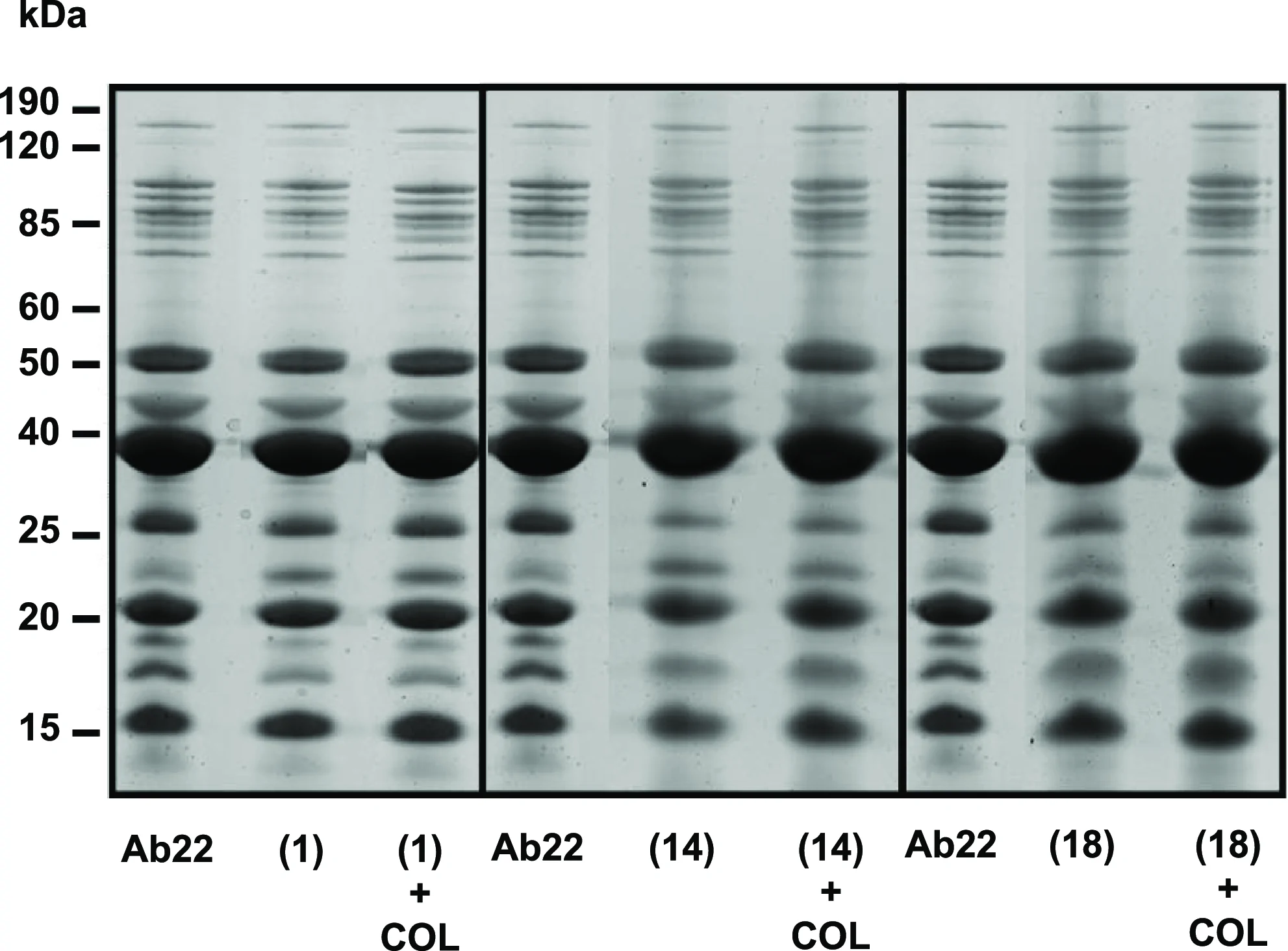
Outer membrane profile of clinical colistin-resistant *A. baumannii* after treatment with tamoxifen derivatives **1**, **14** and **18**. SDS-PAGE of the outer membrane proteins of *A. baumannii* without or with tamoxifen derivatives **1**, **14** and **18** treatments. CTL: control. *A. baumannii Ab22 strain was treated with tamoxifen derivatives **1**, **14** and **18**. COL: colistin.

It is worth noting that a strain-specific effect was observed in this study. This effect implies that the activity of the tamoxifen derivatives in combination with colistin is not uniformly driven by a single, conserved mechanism across *A. baumannii* isolates but is instead modulated by intrinsic bacterial variability. Potential predictors of this response could include genetic markers of membrane modification pathways, OMP expression profiles, and/or strain-specific metabolic or stress-response states. Future omics analyses are needed to confirm whether the observed synergy is not purely drug-driven but instead emerges from a drug-pathogen interaction landscape. In addition, the variability in the tamoxifen derivatives activity may limit the broad clinical applicability of these compounds in their current form. Further structural optimization and evaluation against a larger, genetically diverse collection of clinical isolates will be necessary to better define their therapeutic potential.

## Conclusion

In conclusion, this study provides new insights into the potential use of tamoxifen derivatives against a colistin-resistant *A. baumannii*, a pathogen for which therapeutic options remain severely limited. SAR studies revealed a crucial role of stereochemistry and the type of substituents on the tamoxifen scaffold in determining the synergistic interaction with colistin against the pathogen. Moreover, in the presence of colistin, the active derivatives increased membrane permeability, consistent with previous evidence of the membrane-disruptive effects of tamoxifen metabolites.^10,11^ Further research is required to elucidate their mechanism of action and to optimize dosing strategies for the treatment of severe infections caused by these multidrug-resistant Gram-negative bacteria.

## Materials and Methods

### Bacterial Strains

A total of seven clinical isolates of *A. baumannii* resistant to colistin and one wild-type strain of *A. baumannii* ATCC 17978 were used in this study. All the reported strains were isolated from the University Hospital of Virgen del Rocío in Seville, Spain.^34,35^

### Antimicrobial Agent and Tamoxifen Derivatives

The small library of tamoxifen derivatives was prepared as described in literature, starting from appropriately substituted benzophenones which underwent McMurry olefination.^36,37^ The structures of the tested compounds are presented in Table 1. Standard laboratory powder of colistin (Sigma, Spain) was used also.

### *In Vitro* Susceptibility Testing

The MICs of the tamoxifen derivatives, tested alone and in combination with colistin, were determined against wild-type and colistin-resistant *A. baumannii* strains in two independent experiments using the broth microdilution method, in accordance with the European Committee on Antimicrobial Susceptibility Testing (EUCAST) guidelines.^38^ A standard inoculum of 5 × 10^5^ CFU/mL of each strain, prepared in Luria–Bertani (LB) medium, was added to U-bottom microtiter plates (Deltalab, Spain)^38^ containing the tested tamoxifen derivatives, either alone or in combination with sub-MIC concentrations of colistin (0.125–0.25 mg/L), which did not inhibit bacterial growth.

The interaction between colistin and the tamoxifen derivatives was evaluated in two independent experiments using the FIC index based on the MIC values obtained from the microdilution assays.^24^ Synergy was defined as an FIC index ≤0.5, indicating that the combination significantly enhances the activity of colistin beyond a purely additive effect.^24^

The colistin concentrations used were substantially lower than the corresponding MICs for both the colistin-resistant *A. baumannii* isolates and the reference strain *A. baumannii* ATCC 17978. The plates were incubated at 37 °C for 18 h, and *E. coli* ATCC 25922 was included as a positive control strain.^34^

### Bacterial Growth Curves

To assess antibacterial activity and synergistic effects, bacterial growth curves were obtained in duplicate for the *A. baumannii* Ab22 strain. Overnight cultures grown in LB medium at 37 °C with continuous shaking at 180 rpm were diluted 1:200 in fresh LB medium and transferred to 96-well plates containing compounds **1**, **14**, or **18** (4 mg/L), a sub-MIC concentration of colistin (0.125 mg/L), or the respective combinations. Drug-free broth was included as a growth control. Bacterial growth was monitored for 24 h by measuring the absorbance at 600 nm every 20 min using a Tecan spectrophotometer (model XYZ-2000, Austria).

### Membrane Permeability Assays

Bacterial cells were grown in LB broth and incubated for 3 h in the absence or presence of tamoxifen derivatives **1**, **14**, or **18** (4 mg/L), a sub-MIC concentration of colistin (0.125 mg/L), or their respective combinations. The cells were harvested by centrifugation at 4600*g* for 15 min, washed with 1× PBS, and centrifuged again under the same conditions. The resulting pellet was resuspended in 100 μL of 1× PBS containing 10 μL of ethidium homodimer-1 (excitation/emission wavelengths: 535/624 nm; Thermo Fisher Scientific, USA) and incubated for 10 min. Subsequently, 100 μL of the suspension was transferred to a 96-well plate, and fluorescence was monitored for 300 min using a Typhoon FLA 9000 laser scanner (GE Healthcare Life Sciences, USA). Fluorescence intensity was quantified using ImageQuant TL software (GE Healthcare Life Sciences, USA).^15^

### Analysis of Outer Membrane Proteins

To assess the effects of tamoxifen derivatives on outer membrane proteins (OMPs), cells of the colistin-resistant *A. baumannii* Ab22 strain were grown in LB broth to the logarithmic phase and subsequently treated for 12 h with tamoxifen derivatives **1**, **14**, or **18** (4 mg/L), either alone or in combination with a sub-MIC concentration of colistin (0.125 mg/L). The cells were then lysed by sonication, and OMPs were extracted using sodium lauroyl sarcosinate (Merck, Spain) and recovered by ultracentrifugation, as previously described.^5^ The extracted OMPs were separated by sodium dodecyl sulfate–polyacrylamide gel electrophoresis (SDS-PAGE) on 10% gels, with 6 μg of OMPs loaded per lane. The gels were stained with SimplyBlue SafeStain (Thermo Fisher Scientific, Spain) to visualize the OMP profiles.

### Human Cell Culture

Human type II alveolar epithelial cells (A549) were cultured in DMEM supplemented with 10% heat-inactivated fetal bovine serum (FBS), vancomycin (50 mg/L), gentamicin (20 mg/L), amphotericin B (0.25 mg/L; Invitrogen, Spain), and 1% HEPES at 37 °C in a humidified atmosphere containing 5% CO_2_. The cells were routinely passaged every 3–4 days. Immediately before treatment, the A549 cells were washed three times with prewarmed PBS and subsequently incubated in FBS- and antibiotic-free DMEM.^39^

### Cellular Toxicity

A549 cells were incubated with tamoxifen derivatives **1**, **14** or **18** at concentrations ranging from 0.125 to 2 mg/L for 24 h at 37 °C in a humidified atmosphere containing 5% CO_2_. Before assessing cytotoxicity, the cells were washed three times with prewarmed 1× PBS. Cell viability was then evaluated by measuring mitochondrial metabolic activity using the 3-(4,5-dimethylthiazol-2-yl)-2,5-diphenyltetrazolium bromide (MTT) assay as described previously.^40^ The percentage of cytotoxicity was calculated from the absorbance at 570 nm as follows

[(absorbance570nm⁢of⁢treatedcells/absorbance570nmmean⁢ofun⁢treatedcells)×100]

### Statistical Analysis

Group data are presented as means ± standard errors of the means (SEM). Student’s *t*-test was used to determine differences between means using the GraphPad Prism 9. A *p*-value <0.05 was considered significant.
